# Complete Genome Sequence of *Bacillus amyloliquefaciens* Strain Co1-6, a Plant Growth-Promoting Rhizobacterium of *Calendula officinalis*

**DOI:** 10.1128/genomeA.00862-15

**Published:** 2015-08-13

**Authors:** Martina Köberl, Richard A. White, Sabine Erschen, Nora Spanberger, Tarek F. El-Arabi, Janet K. Jansson, Gabriele Berg

**Affiliations:** a Graz University of Technology, Institute of Environmental Biotechnology, Graz, Austria; b Pacific Northwest National Laboratory, Biological Sciences Division, Richland, Washington, USA; c University of Graz, Institute of Plant Sciences, Graz, Austria; d Ain Shams University, Faculty of Agriculture, Cairo, Egypt; e Heliopolis University, Biotechnology Laboratory, Cairo, Egypt

## Abstract

The genome sequence of *Bacillus amyloliquefaciens* strain Co1-6, a plant growth-promoting rhizobacterium (PGPR) with broad-spectrum antagonistic activity against plant-pathogenic fungi, bacteria, and nematodes, consists of a single 3.9-Mb circular chromosome. The genome reveals genes putatively responsible for its promising biocontrol and PGP properties.

## GENOME ANNOUNCEMENT

*Bacillus amyloliquefaciens* Co1-6 was isolated in October 2009 from the rhizosphere of the pot marigold *Calendula officinalis* L., cultivated on the organically managed Sekem farms in the northeastern desert region of Egypt (30°22′88″N 31°39′41″E) (1). The soil texture at the desert farm was classified as loamy sand, with a clay content of 4%, organic carbon content of 0.8%, and alkaline pH of 8.4 (2). Co1-6 was selected as a broad-spectrum antagonist exhibiting antifungal (*Verticillium dahliae*, *Rhizoctonia solani*, and *Fusarium culmorum*), antibacterial (*Ralstonia solanacearum*), and nematicidal (*Meloidogyne incognita*) activity against soilborne phytopathogens (3). For strains of the same population, induced systemic resistance of the host plant was identified as the major reason for their nematicidal activity (4). Treatment of chamomile plants (*Matricaria chamomilla*) with Co1-6 under field conditions resulted in elevated flavonoid contents of the blossoms (5).

Genomic DNA was extracted using the MasterPure DNA purification kit (Epicentre, Madison, WI, USA), modified with additional cell disruption steps comprising mechanical shredding with glass beads in a FastPrep instrument (MP Biomedicals, Santa Ana, CA, USA) and lysozyme-based cell wall digestion. PacBio RS libraries with inserts of 8 to 20 kb were constructed and sequenced at GATC Biotech (Konstanz, Germany).

Whole-genome shotgun sequencing yielded 245,374 raw reads with 1,486,144,876 bp of raw sequence. Assembly was completed with the Hierarchical Genome Assembly Process (HGAP) algorithm implemented in the PacBio SMRT Analysis software (Pacific Biosciences, Menlo Park, CA, USA) and resulted in a single circular chromosome of 3,922,431 bp, with 378.9-fold overall coverage and a G+C content of 46.85%.

The closest relative of Co1-6 based on the full-length 16S rRNA gene sequence is *B. amyloliquefaciens* subsp. *plantarum* FZB42 (NCBI reference sequence no. NR_075005, 99% sequence similarity). FZB42 is a well-known PGPR serving as the basis of a commercially available product (RhizoVital 42; ABiTEP GmbH, Berlin, Germany) with the ability to stimulate plant growth and suppress plant pathogens (6). Digital DNA-DNA hybridization (DDH) using GGDC 2.0 (7–9) against the genome sequence of FZB42 (accession no. NC_009725) estimated a DDH of 80.30% ± 2.77%, indicating that they have 90.8% probability of being the same species but only 48.3% probability of being the same subspecies.

Annotation was conducted on the RAST Web server using RAST gene calling based on FIGfam version Release70 (10, 11), and additional annotation was completed on the BASys Web server using Glimmer gene prediction (12, 13). The genome annotation contained 3,913 predicted protein-coding genes, 86 tRNA and 19 rRNA loci, and 457 predicted SEED subsystem features.

The genome encodes synthases for mycosubtilin, plipastatin, and surfactin antibiotics, which most probably contribute to the promising abilities of Co1-6 for pathogen suppression. Co1-6 revealed six additional polyketide synthases, some at up to seven copies, and a dimodular nonribosomal peptide synthase. We further identified genes most probably involved in the direct promotion of plant growth, such as biosynthesis gene clusters for rhizobactin siderophores, spermidine, and auxin.

### Nucleotide sequence accession numbers.

This whole-genome shotgun project has been deposited in the European Nucleotide Archive under the accession no. CVPA00000000. The version described in this paper is the first version, CVPA01000000.
